# Comparative outcomes of primary ureteral reimplantation vs. staged cutaneous ureterostomy in infants under one with primary obstructive megaureters and vesicoureteral reflux: a multi-center analysis

**DOI:** 10.1007/s00383-025-06220-6

**Published:** 2025-10-16

**Authors:** Moayad Beibooh, Jawdat Jaber, Galiya Raisin, Boris Chertin, Stanislav Kocherov, Leon Chertin

**Affiliations:** 1 The Department of Pediatric Urology, Affiliated with the Faculty of Medicine, Shaare Zedek Medical Center, The Hebrew University, Jerusalem, Israel; 2 Department of Urology, Shamir Medical Center, Zerifin, Israel; 3 Affiliated to the Sackler School of Medicine, Tel-Aviv University, Tel-Aviv, Israel

**Keywords:** Antenatal urinary tract dilatation, Primary obstructive megaureter, Vesicoureteral reflux, Ureteral reimplantation, Cutaneous ureterostomy, Pediatric urology

## Abstract

**Purpose:**

This study evaluates the management and long-term outcomes of pediatric patients under 1 year of age with primary obstructive megaureters (POM) and vesicoureteral reflux (VUR), comparing primary ureteral reimplantation (PR) to a two-stage approach involving cutaneous ureterostomy (CU) followed by reimplantation.

**Methods:**

A multi-institutional study was conducted between 1994 and 2024, including 28 pediatric patients under 12 months of age. Participants were divided into two groups: PR (*n* = 14) and CU (*n* = 14). Comprehensive preoperative assessments, including renal ultrasound, voiding cystourethrography, and radionuclide diuretic renal scans, were performed. Surgical indications were based on recurrent UTIs, impaired renal function, or progressive hydronephrosis.

**Results:**

No significant difference in hydroureteronephrosis grade (SFU) was observed between the two groups (*p* < 0.05). In the PR group, two children required additional surgeries due to recurrent UTIs. Similarly, in the CU group, two children underwent subsequent ureteral reimplantation. The mean age at primary surgery was 6.9 months for the PR group, compared to 4 months for ureterostomy in the CU group (*p* < 0.05). The average operation time was 110.5 min for PR, vs. 64 min for CU (*p* < 0.05). Overall, more complications occurred in the group of children with CU until a definitive repair was performed.

**Conclusions:**

Both primary ureteral reimplantation and the two-stage approach with cutaneous ureterostomy followed by reimplantation demonstrated effective outcomes in managing POM and VUR in infants. Given the findings, primary ureteral reimplantation may be considered a safe and effective approach in infants under 1 year with these conditions.

**Supplementary Information:**

The online version contains supplementary material available at 10.1007/s00383-025-06220-6.

## Introduction

Urinary tract dilatation (UTD) is the second most frequently diagnosed prenatal anomaly, affecting approximately 1–2% of fetuses [1]. The causes of UTD vary, with a transient form occurring in 50–70% of cases and resolving spontaneously over time. Among the known etiologies, ureteropelvic junction obstruction (UPJO) is the most common cause of antenatal UTD, followed by vesicoureteral reflux (VUR) in 10–40% of cases, and ureterovesical junction obstruction (UVJO) in 5–15% of cases [1]. Obstructive megaureter is defined as a ureter diameter exceeding 7 mm. For children with grade III–V VUR, the European Association of Urology (EAU) guidelines recommend initial antibiotic prophylaxis. However, for those with high-grade VUR, surgical options such as ureteral reimplantation should be considered [2]. In symptomatic patients, clinical signs such as pain, recurrent urinary tract infections (UTIs) despite antibiotic prophylaxis, or worsening UTD, along with a differential renal function below 40% or a progressive decline on serial renograms, may indicate the need for surgical intervention [3]. A range of treatment options are available, with the choice influenced by the child’s overall health, age, presence of other malformations, and the severity of the condition [1]. Depending on VUR severity, minimally invasive procedures like endoscopic correction can be considered. We have previously described our experience in the treatment of more than 1300 reimplantations due to VUR and primary obstructive megaureter (POM) [4]. In cases requiring surgery during infancy, the approach may involve primary ureteral reimplantation (PR) or a staged procedure, which includes an initial cutaneous ureterostomy (CU) followed by delayed reimplantation. The rationale for the two-stage procedure is to reduce the complexity and duration of the first surgery in infancy. The British Association of Pediatric Urologists suggests deferring definitive repair until the child reaches 1 year of age, using temporizing interventions in the meantime [3]. However, this recommendation lacks strong supporting evidence and exposes the child to additional procedures. CU is associated with risks, such as stomal stenosis (8–22%), febrile UTIs (31%), and pyelonephritis [3]. Few studies have directly compared the safety, efficacy, and long-term outcomes of primary definitive repair vs. a staged approach in infants under 12 months with POM [5].

In this multicenter study, we aim to evaluate the management and long-term outcomes of pediatric patients under 1 year of age with POM and VUR who underwent either PR or a two-stage procedure (CU followed by reimplantation). Our hypothesis is that PR is a safe and effective treatment in infancy compared to the staged approach.

## Materials and methods

This multi-institutional study includes all pediatric patients under 12 months underwent either PR or CU for POM or VUR between 1994 and 2024 across two medical centers, with ethical approval from the respective institutional review boards of both centers. Perioperative and follow-up data were meticulously collected and are presented in Table 1. Perioperative and follow-up data were meticulously collected for each patient. The preoperative assessment included a comprehensive history, physical examination, urinalysis, urine culture, renal ultrasound (US), radionuclide diuretic renal scan (MAG3), and voiding cystourethrography (VCUG). Surgical indications for the POM group were based on the British Association of Pediatric Urologists (BAPU) consensus guidelines and included recurrent UTIs, impaired renal function (with a relative function of less than 40% on MAG3 scans), deterioration of function (greater than 10% worsening on consecutive MAG3 scans), or progressive hydronephrosis and/or hydroureter [3]. For the VUR group, indications for surgery included recurrent febrile UTIs despite prophylactic antibiotics, or evidence of obstructive refluxing megaureter supported by existence of beak sign on the VCUG images accompanied by deterioration of the renal function or break through infections while on antibiotic prophylaxis. Postoperatively all patients have had US examinations between 3 and 6 months postoperatively. Obligatory MAG 3 radionuclide scan was reserved for those with deterioration of hydronephrosis. Those who have demonstrated stable or improvement of hydronephrosis were scheduled for follow-up US examinations only, unless parents or primary physician were asked for radionuclide scan. We have calculated the fractional left and right renal activity for each kidney. A kidney uptake of 40 to 50% of total renal activity was considered as normal. Relative renal function of 30–40% was considered as moderate and renal function below 30% of relative renal function was considered as poor. VCUG was recommended for these patients who presented with febrile UTI during follow-up or those who have had Rec Low UTI to rule out reflux correction failure or appearance of the de novo reflux.

**Table 1:** Demographic variables of the participants

No	PR (n = 14)	CU (n = 14)
Demography and clinical details		
Antenatal detection	12/14 (86%)	13/14 (93%)
Average gestational age	39	39
Sex distribution	M—10 (71%), F—4 (29%)	M—13 (93%), F-1 (7%)
Side laterality	LT—10 (71%), RT-3 (21%), Bilateral 1 (7%)	LT—8 (57%), RT-6 (43%)
Etiology(possibly combined)	10 UVJS, 5 OMU, 3 VUR,	10 UVJS, 8 OMU, 1 PUV
	3 POMU	3 VUR, 1 POMU
Other urogenital anomalies	8/14 (57%)	7/14 (50%)
Clinical symptoms		
UTI	4	2
AKI	2	2
Asymptomatic/unknown	8	10
Prophylactic antibiotic—preoperative	Yes—8 (57%), no—5 (36%), Unknown—1 (7%)	yes—12 (86%), no—2 (14%)

### Surgical techniques for ureteral reimplantation

#### Open ureteral reimplantation

The LEADBETTER–POLITANO method was utilized in our institutions. The affected ureter is mobilized and electrically dissected intravesically. A submucosal tunnel is created, and the ureter is fixed to the bladder muscle after slight shortening.

#### Robotic-assisted laparoscopic ureteral reimplantation (RALUR)

Using the Da Vinci Xi platform, a transperitoneal approach is employed, following the Lich–Gregoir method [6, 7]. The patient is positioned supine, and general anesthesia is induced, often with caudal or quadratus lumborum blocks [8]. The procedure begins with trocar placement (8-mm camera port, two 8-mm working ports). The distal ureter is dissected to the UVJ with particular attention to avoiding damage to pelvic nerve branches. Once the tunnel is created, the ureter is placed into the detrusor muscle, and the tunnel is sutured over it. Special care is taken to avoid injury to the vas deferens. No external drains or prophylactic antibiotics are used postoperatively.

#### Robotic-assisted dismembered extravesical cross-trigonal ureteral reimplantation (RADECUR)

This technique is used for complex cases as we previously described [4]. A transverse detrusorrhaphy is performed on the ipsilateral side of the contralateral ureter. The ureter is tailored when needed, and a bladder–ureter anastomosis is created using Maxon 4–0 sutures over a double-J stent. Detrusorrhaphy is closed with a running V-Lock suture.

#### Delayed staged repair (DSR)

Infants undergo CU to drain the obstructed system. Extraperitoneal exposure is achieved via a modified Gibson incision. After transecting the ureter, a lateral ureteral stoma is created, which is left intubated during the early postoperative course. After a year, a definitive repair with intravesical reimplantation is performed. The urethral catheter is removed on day 1. In those children who required bilateral CU bladder cycling program was initiated following procedure to avoid dry bladder and allow normal bladder capacity development and continued till definitive treatment.

### Statistical analysis

Categorical data were analyzed using the Fisher exact or chi-square test, with significance at *p* < 0.05. Statistical evaluations were conducted using GraphPad Prism version 6.01.

## Results

Twenty-eight children with UTD of varying etiology were included in this study. Fourteen underwent PR, and fourteen underwent CU as part of a two-stage approach. Operative and postoperative data are presented in Table 2. In brief, both preoperative groups had a high proportion of severe hydronephrosis, as classified by the Society for Fetal Urology (SFU) grading system, with the CU group having slightly more severe cases 9 SFU IV und 4 SFU III vs. 7 SFU IV und 3 SFU III in the PR group. At 1-year follow-up both groups showed improvement, with many patients downgraded to SFU I or II. In addition, both groups had patients with normal US findings postoperatively (1 in the PR group and 2 in the CU group).

**Table 2:** Surgical and post-surgical variables of the participants

No	PR (n = 14)	CU (n = 14)
Operative details		
Mean age at surgery	6.9 (3–11 months)	4 (2 days–6 months)
Surgery mode	Open—12, robotic—2	open—13, robotic—1
Ureteral Tapering	May-14	–
Average OP-Time (min.)	110.5 (80–192)*	64 (34–115)
Post-operative follow-up		
Average hospital stay (days)	6.4 (1–16)	4.1 (1–25)
Postop. complications (30 days)		
UTI	1/14	0/14
SFU/VUR Grading		
Pre-operative	7 SFU IV, 3 SFU III (all VUR IV), 4 unknown	1 SFU II, 4 SFU III, 9 SFU IV (incl. VUR V)
At 1-year follow-up	2 SFU I, 4 SFU II, 1 normal US (VUR), 1 SFU III (VUR IV), 1 SFU 4, 5 unknown**	4 SFU I, 5 SFU II*, 2 SFU III 2 normal US (1 VUR), 1 unknown
Postoperative recurrent UTI (> 2) (> 30 days after surgery)	2/14	4/14
Postoperative long term prophylactic antibiotics	2/14	5/14
Following/Redo Operation	2/14	0/14
Success rate	86%	86%
Definitive repair after ureterostomy		
Average Time to 2. OP	–	16.6 (2–24) months
OP mode	–	7/14 open, 4/14 robotic,
		1/14 Nephrectomy, 2/14 not yet

*Include the total op-time of 2 combined operations (PR + pyeloplasty/PR + Drainage of paraureteral diverticula)

**Unknown or not completed 1-year follow-up

After PR, we observed a significant reduction in SFU grade in our patient cohort at the 1-year follow-up (*p* < 0.05). Two children developed recurrent UTIs due to underlying persistent VUR despite prophylactic antibiotics, requiring two additional surgeries in the form of endoscopic VUR correction. Similarly, after CU, we observed a significant reduction in SFU grade at 1-year follow-up (*p* < 0.05). In two children, moderate–severe hydronephrosis was still detectable postoperatively. One child with recurrent UTIs despite prophylactic antibiotic treatment was found to have an additional UVJ stenosis on the contralateral side and underwent bilateral open ureter reimplantation at the age of 3. The second child developed two episodes of idiopathic urinary retention. Subsequently, an open ureteral reimplantation was performed due to de novo VUR on the contralateral side. When comparing the SFU grades between the two groups, no statistically significant difference was found both preoperatively and at 1 year postoperatively. In the PR group, ureteral tapering was performed in 5/14 cases, compared to none in the comparison group. Ureteral diameter was documented only in isolated cases, so we decided not to conduct further analysis on this parameter. Surgery in the PR group was performed at a mean age of 6.9 months, compared to 4 months in the CU group (*p* < 0.05). Regarding the duration of operation, the procedure in the PR group took an average of 110.5 min, compared to 64 min in the CU group (*p* < 0.05). In addition, there was a statistically significant difference in the mean hospital stay, with 6.4 days in the PR group vs. 4.1 days in the CU group (*p* < 0.05). A postnatal impaired kidney function (abnormal creatinine, GFR was not routinely estimated) was observed in two children in each group. In the case of one child in each group, the kidney function normalized after hydration, allowing the surgery to be delayed and performed electively. The second child in the PR group had a single kidney with POM and UPJS. Initially, a nephrostomy was placed postnatally. The kidney function improved after the definitive surgery at age 7 months but did not normalize. In contrast, the second child in the CU group had a PUV with a single functioning kidney. Severe AKI was diagnosed on the second day of life, so surgery was promptly performed on the same day. Regarding associated urogenital anomalies, 4/14 children in the PR group had UPJS. The child with the longest operation time in this group (192 min.) underwent a combined procedure of robotic ureter reimplantation and pyeloplasty. Two children in the PR group underwent PR 5 and 2 months after pyeloplasty, respectively. One child was diagnosed with UPJS after the PR surgery. Three children had a single “functioning” kidney. A para-ureteral diverticulum was simultaneously operated on in one child with PR. Other anomalies included VUR I on the contralateral side and cross-right kidney with ectopic right ureter. In the CU group, 3/14 children had UDT, and 2/14 had a single “functioning” kidney. In addition to the above-mentioned child with PUV in this group, two other children were diagnosed with PUV postnatally and were treated with fulguration as neonates. With regard to postoperative complications (< 30-day post-surgery), one child in each group was diagnosed with a UTI, with a positive urine culture. In the further postoperative course, we considered recurrent UTIs in the PR group in the two aforementioned children with persistently postoperative VUR. In the CU group, in addition to the aforementioned child considered a surgical failure, recurrent UTIs were observed in three other children. Two children were definitively treated, and the other child has not yet undergone definitive surgery. In general, the indication for long-term postoperative (after PR or CU) antibiotic treatment was the presence of recurrent UTIs (6/7 children: 2/2 in the PR group and 4/5 in the CU group) or in children with persistent hydroureteronephrosis and pending definitive closure (1/5 in the CU group). By January 2025, definitive therapy had been performed in 11/14 children in the CU group. The average time between the two surgeries was 16.6 months. In one child, a nephrectomy of the affected kidney was performed due to poor function. This child had an initial postnatal partial renal function (PRF) of 16%. After the CU, the kidney did not recover, and the PRF deteriorated to 8%. Surgery is still pending in two children.

The average follow-up duration was 33 months in the PR group and 89.5 months in the CU group. This is partly due to some infants in the PR group having a relatively short follow-up period. Furthermore, the infants in the CU group logically require a longer follow-up period due to the staged repair and the time it takes to achieve a definitive repair.

## Discussion

The management of UTD in infants is complex and challenging in pediatric urology [9]. Compared to treatment protocols for children over the age of one, the optimal therapeutic approach for infants under 1 year of age is notably more intricate, with limited high-quality evidence guiding clinical decision-making [5]. Few studies have directly compared postoperative outcomes for infants, and most are retrospective cohort analyses lacking adequate control groups [10]. Patient population heterogeneity complicates definitive conclusions, as many studies include infants with diverse underlying conditions [1]. The British Association of Pediatric Urology recommends PR for children older than 1 year, though this does not apply to infants [3]. Some studies challenge this by demonstrating favorable outcomes for PR in infants, with one study showing a 97% success rate for infants compared to 86% in older children [11]. In addition, no significant difference in bladder function was observed between infants under 12 months and children aged 1–10 years following PR [12]. In this study, both groups had a success rate of 87%, aligning with studies like Patil et al., who reported 94.4% [5] and others that showed success rates of 92–100% for PR in infants [10, 11, 14–17]. This study found a male predominance and left-sided laterality, consistent with other studies [5, 11]. Most cases were diagnosed prenatally, with only 6 out of 28 diagnosed after UTI, contrasting with other studies that report higher UTI rates [5, 17]. The mean age at surgery was 6.4 months for the PR group and 4 months for the CU group, similar to Patil et al. [5]. Other studies report PR can be done as early as 1.8–4 months [10, 13]. Ureteral tapering during PR was done in 36% of cases in this study, lower than the 65–77% reported elsewhere [5, 10]. Most surgeries were open, with only a few robot-assisted cases. The average operative time for PR was 110.5 min, and for CU, it was 64 min, similar to Patil et al. [5]. A meta-analysis found no significant difference between robotic and open PR in success rates or complications, though open PR had shorter operative times but longer hospital stays [22]. In this study, the average length of stay was 6.4 days for PR and 4.1 days for CU. These figures differ from others, with studies showing shorter stays for younger infants undergoing PR [11, 16]. Hydronephrosis severity decreased significantly at the 1-year follow-up, consistent with previous studies [5]. The higher number of unknowns in the PR group may limit direct comparison. In addition, we have not observed deterioration of the renal function in those patients who underwent postoperative radionuclide study according to our protocol. Although in part improvement in renal function might be associated with physiological glomerular maturation altogether with improvement of the SFU it supports the safety and efficacy of our approach. The prevalence of associated urogenital malformations in our cohort was higher (50–57%) compared to other studies, which reported 20.2–23.3% [23]. Postoperative complications within 30 days included one UTI in each group (7%), lower than other studies reporting 15% for PR in infants [11]. Interestingly, while Patil et al. reported a relatively high complication rate of 25% following CU, and Kitchens documented a 31% UTI rate post-CU, this study record only one UTI as a short term CU-related complication [5, 24]. Nevertheless, in the CU group, the rate of UTIs and UTI-related hospital admissions was higher. In addition, the CU requires a certain level of specialized and qualified care. The skin irritations present in the majority of patients in the CU group were not pursued as complications in our clinic due to their mild severity. These are additional factors that speak against CU.

This study is not without limitations. The retrospective nature of our analysis introduces potential biases, including selection bias and variability in surgical decision-making. We could not comment on the postoperative changes in the ureteral diameter following surgical correction, since these data were not recorded in all patients; however, we have demonstrated in overall improvement of SFU hydronephrosis grade in our patients following successful surgery. We also could not comment on the overall changes in the renal function following surgery, since not all patients underwent renal scan before and after definitive surgery as we have mentioned. However, all patients who demonstrated deterioration of hydronephrosis showed stable renal function after surgery, and those who chose to go ahead with renal scan in spite improvement of hydronephrosis demonstrated either improved or stable renal function. The relatively small sample size limits the generalizability of our findings. In addition, the heterogeneity of our patient population, including mixed etiologies and varied severity of disease, complicates direct comparisons with other studies. Furthermore, we could not make a direct comparison between the patients with POM vs. VUR. The numbers are small, so the meaningful conclusion is not possible. Moreover, it is well-known that some of the high grade VUR patients presented with unfunctional and non-peristaltic obstructive component in the UVJ making homogeneous subgroup comparison impossible. Overall, our findings contribute to the growing body of literature on the surgical management of UTD in infants, highlighting comparable success rates between PR and CU while also shedding light on differences in complication rates, hospital stay duration, and associated anomalies. As evidence continues to evolve, future studies incorporating larger, well-controlled patient cohorts will be essential in refining treatment guidelines and optimizing surgical outcomes for this vulnerable population. It is worth mentioning that robotic approaches evolving in pediatric population and the place of laparoscopic robotic assisted reimplantation in the children younger than 1 year should be carefully evaluated.

In Conclusion, PR in infants is a safe and effective treatment option, comparable to CU. As a single-stage procedure, PR eliminates the need for additional surgeries, reducing the overall treatment burden on patients. In addition, it helps prevent complications associated with urostomies. The increasing adoption of robotic-assisted PR has further improved outcomes, allowing for quicker postoperative recovery and shorter hospital stays, making it a viable and efficient approach for managing urinary tract dilation in infants.

## Supplementary Information

Below is the link to the electronic supplementary material.

Supplementary file1 (DOCX 23 KB)

Supplementary file2 (DOCX 30 KB)

## Data Availability

No data sets were generated or analysed during the current study.
