# Supplementary material for: Comparative outcomes of primary ureteral reimplantation vs. staged cutaneous ureterostomy in infants under one with primary obstructive megaureters and vesicoureteral reflux: a multi-center analysis
Source: Pediatr Surg Int. 2025 Oct 16;41(1):316. doi: 10.1007/s00383-025-06220-6 (PMC12532621; doi:10.1007/s00383-025-06220-6)
Supplement: Supplementary file 1 — Supplementary file1 (DOCX 23 KB) [file 383_2025_6220_MOESM1_ESM.docx]

**Table 1** Demographic variables of the participants

PR (n=14) CU (n=14)

**Demography and clinical details**

Antenatal detection 12/14 (86%) 13/14 (93%)

Average gestational age 39 39

Sex distribution M – 10 (71%), F - 4 (29%) M - 13 (93%), F-1 (7%)

Side laterality LT – 10 (71%), RT- 3 (21%), LT – 8 (57%), RT- 6 (43%)

Bilateral 1 (7%)

Etiology (possibly combined) 10 UVJS, 5 OMU, 3 VUR, 10 UVJS, 8 OMU, 1 PUV

3 POMU 3 VUR, 1 POMU

Other urogenital anomalies 8/14 (57%) 7/14 (50%)

Clinical symptoms

- UTI 4 2
- AKI 2 2
- Asymptomatic/unknown 8 10

Prophylactic antibiotic - preoperative yes – 8 (57%), no – 5 (36%), yes - 12 (86%), no - 2 (14%)

Unknown - 1 (7%)
