# Supplementary material for: Comparative outcomes of primary ureteral reimplantation vs. staged cutaneous ureterostomy in infants under one with primary obstructive megaureters and vesicoureteral reflux: a multi-center analysis
Source: Pediatr Surg Int. 2025 Oct 16;41(1):316. doi: 10.1007/s00383-025-06220-6 (PMC12532621; doi:10.1007/s00383-025-06220-6)
Supplement: Supplementary file 2 — Supplementary file2 (DOCX 30 KB) [file 383_2025_6220_MOESM2_ESM.docx]

**Table 2** Surgical and post-surgical variables of the participants

PR (n=14) CU (n=14)

**Operative details**

Mean age at surgery 6.9 (3-11 months) 4 (2 days-6 months)

Surgery mode open – 12, robotic – 2 open – 13, robotic - 1

Ureteral Tapering 5/14 -----

Average OP-Time (min.) 110.5 (80-192)* 64 (34-115)

**Post operative follow up**

Average hospital stay (days) 6.4 (1-16) 4.1 (1-25)

Postop. complications (30 days)

- UTI 1/14 0/14

SFU/VUR Grading

- Preoperative 7 SFU IV, 3 SFU III (all VUR IV), 1 SFU II, 4 SFU III,

4 unknown 9 SFU IV (incl. VUR V)

- At 1^st^ year follow up 2 SFU I, 4 SFU II, 1 normal US (VUR), 4 SFU I, 5 SFU II*, 2 SFU III

1 SFU III (VUR IV), 1 SFU 4, 2 normal US (1 VUR),

5 unknown** 1 unknown**

**Renal function %**

- **Preoperative**

**Normal (40-50) 1**

**Moderate (30-40) 8 7**

**Poor/Non-functioning (<30) 1 2**

- **At 1^st^ year follow up**

**Normal (40-50) 1 1**

**Moderate (30-40) 6 5**

**Poor/Non-functioning (<30) 1 1**

postoperative recurrent UTI (>2) 2/14 4/14

(>30 days after surgery)

Postoperative long term 2/14 5/14

prophylactic antibiotics

Following/Redo Operation 2/14 0/14

Success rate 86% 86%

**Definitive repair after ureterostomy**

Average Time to 2. OP ----- 16.6 (2-24) months

OP mode ----- 7/14 open, 4/14 robotic,

1/14 Nephrectomy, 2/14 not yet

* include the total op-time of 2 combined operations (PR+pyeloplasty/PR+ Drainage of paraureteral diverticula)

** unknown or not completed one year follow up
